# Absence of BCL-2 Expression Identifies a Subgroup of AML with Distinct Phenotypic, Molecular, and Clinical Characteristics

**DOI:** 10.3390/jcm9103090

**Published:** 2020-09-25

**Authors:** Inke De haes, Amélie Dendooven, Marie Le Mercier, Pauline Puylaert, Katrien Vermeulen, Mark Kockx, Kathleen Deiteren, Marie-Berthe Maes, Zwi Berneman, Sébastien Anguille

**Affiliations:** 1 Division of Hematology, Antwerp University Hospital, 2650 Edegem, Belgium; inke.dehaes@student.uantwerpen.be (I.D.h.); zwi.berneman@uza.be (Z.B.); 2 Division of Pathology, Antwerp University Hospital, 2650 Edegem, Belgium; amelie.dendooven@uza.be; 3 Division of Pathology, Ghent University Hospital, 9000 Ghent, Belgium; 4 Division of Clinical Biology, Antwerp University Hospital, 2650 Edegem, Belgium; marie.lemercier@uza.be (M.L.M.); katrien.vermeulen@uza.be (K.V.); kathleen.deiteren@uza.be (K.D.); marie-berthe.maes@uza.be (M.-B.M.); 5 Laboratory of Physiopharmacology, University of Antwerp, 2610 Wilrijk, Belgium; pauline.puylaert@uantwerpen.be; 6 HistoGeneX, 2610 Wilrijk, Belgium; mark.kockx@histogenex.com; 7 Laboratory of Experimental Hematology, Vaccine & Infectious Disease Institute (VAXINFECTIO), Faculty of Medicine & Health Sciences, University of Antwerp, 2610 Wilrijk, Belgium

**Keywords:** acute myeloid leukemia, immunohistochemistry, BCL-2, venetoclax, *FLT3*, next-generation sequencing

## Abstract

Acute myeloid leukemia (AML) is a hematologic malignancy characterized by the rapid and uncontrolled clonal growth of myeloid lineage cells in the bone marrow. The advent of oral, selective inhibitors of the B-cell leukemia/lymphoma-2 (BCL-2) apoptosis pathway, such as venetoclax, will likely induce a paradigm shift in the treatment of AML. However, the high cost of this treatment and the risk of additive toxicity when used in combination with standard chemotherapy represent limitations to its use and underscore the need to identify which patients are most—and least—likely to benefit from incorporation of venetoclax into the treatment regimen. Bone marrow specimens from 93 newly diagnosed AML patients were collected in this study and evaluated for BCL-2 protein expression by immunohistochemistry. Using this low-cost, easily, and readily applicable analysis method, we found that 1 in 5 AML patients can be considered as BCL-2^−^. In addition to a lower bone marrow blast percentage, this group exhibited a favorable molecular profile characterized by lower *WT1* expression and underrepresentation of *FLT3* mutations. As compared to their BCL-2^+^ counterparts, the absence of BCL-2 expression was associated with a favorable response to standard chemotherapy and overall survival, thus potentially precluding the necessity for venetoclax add-on.

## 1. Introduction

Acute myeloid leukemia (AML) is a hematologic malignancy characterized by the rapid and uncontrolled clonal growth of myeloid lineage cells in the bone marrow [1]. The standard frontline treatment for AML comprises a combination of an anthracycline for 3 days and cytarabine for 7 days, with this “3 + 7” intensive chemotherapy (IC) regimen serving as the backbone of AML treatment since the 1970s [2]. During the previous decade, hypomethylating agents (HMAs), such as decitabine and azacitidine, have complemented the treatment armamentarium for AML. These less intensive drugs are predominantly used in elderly AML patients or those who are unfit for IC [3].

Even with IC or HMAs, the outcome of AML remains poor, as exemplified by a 5-year overall survival (OS) rate of 30% [4]. This has led to a search for novel, more effective treatment approaches. One of these approaches is based on the induction of AML cell apoptosis by inhibiting B-cell leukemia/lymphoma-2 (BCL-2) [5]. BCL-2 is an anti-apoptotic protein commonly expressed in hematological malignancies, including AML. In 2018, the US Food and Drug Administration approved the first oral, selective BCL-2 inhibitor (venetoclax) for use in combination with HMAs in newly diagnosed AML patients aged ≥75 years and unfit for IC. The results of the pivotal phase III VIALE-A study investigating the combination of venetoclax and azacitidine were recently published and confirmed an OS advantage in the above population [6]. Studies are currently underway to investigate the added value of venetoclax in AML patients treated with IC [5].

Given the financial burden of venetoclax treatment and the risk of additive toxicity when combined with IC or HMAs, it is critically important to determine which patients will most benefit from the addition of venetoclax [7,8,9,10,11,12,13]. Next-generation sequencing (NGS) is a valuable tool that can be used to identify gene mutations conferring resistance or sensitivity to venetoclax [8,14]. For example, patients harboring *nucleophosmin 1* (*NPM1*) or *isocitrate dehydrogenase* (*IDH*) mutations consistently respond well to combined venetoclax–chemotherapy treatment [9,12,15,16]. Nevertheless, there remain limitations to NGS use, including cost and turnaround time (2–3 weeks), which hampers rapid treatment decisions [17]. Here, we demonstrate that the analysis of BCL-2 protein level can be used as a rapid and low-cost method to identify a subgroup of BCL-2^−^ AML patients with high sensitivity to standard IC or HMAs, thereby precluding the need for adding venetoclax treatment in approximately one in five newly diagnosed AML patients.

## 2. Patients and Methods

### 2.1. Patients

This post-diagnostic study involved all newly diagnosed patients aged ≥18 years with a 2016 World Health Organization (WHO)-confirmed diagnosis of AML [18], diagnosed between 1 January 2017 and 31 December 2019 at the Antwerp University Hospital (Antwerp, Belgium). Patients with acute promyelocytic leukemia were excluded from the study. For each patient, we collected demographic data (age, gender, date of diagnosis, therapy received, and date of remission/relapse/death, if applicable). Patients were risk stratified in three groups (favorable, intermediate, and adverse risk) using the European LeukemiaNet (ELN) 2017 criteria [1] or, if there were insufficient data for ELN classification, using the National Comprehensive Cancer Network cytogenetic risk classification [19].

### 2.2. Routine Laboratory Tests

In addition, the following laboratory test values, which are routinely performed at our center during the diagnostic work-up of AML, were recorded: hemoglobin (g/dL), platelet count (×10^9^/L), white blood cell (WBC) and absolute neutrophil counts (×10^9^/L), peripheral blast count (×10^9^/L; determined by morphology and/or flow cytometry with 1% as the threshold for positivity), bone marrow blast percentage (determined by morphology and/or flow cytometry), presence of CD14, CD34, and CD64 on the bone marrow blast cell population (determined by flow cytometry), HemaVision^®^ multiplex reverse transcriptase (RT) polymerase chain reaction (PCR) results (DNA Diagnostic, Risskov, Denmark), the presence of internal tandem duplications (ITDs) and tyrosine kinase domain (TKD, codons 835 and 836) mutations in the *fms-like tyrosine kinase 3* (*FLT3*) gene (determined by standard PCR fragment analysis), peripheral blood and bone marrow *Wilms’ tumor 1* (*WT1*) transcript levels (determined as per our previously described methodology [20,21]), and conventional cytogenetic data.

### 2.3. BCL-2 Immunohistochemistry

Bone marrow core biopsies obtained at diagnosis were collected and analyzed for BCL-2 protein expression by immunohistochemistry (IHC), which was performed on a Dako Omnis platform (Agilent Technologies, Santa Clara, CA, USA) using the corresponding anti-BCL-2 antibody (clone 124). The percentage of BCL-2-expressing blast cells was manually scored under a light microscope by two independent observers (I.D.H. and A.D.) using the H-method, which is a simple, validated semi-quantitative immunostaining score of intensity and extent [22]. Intensity was graded on a scale of 0 to 3 (0 = absent, 1 = weak, 2 = moderate, and 3 = intense). Extent of staining was scored from 0 to 100, which refers to the percentage of blast cells staining positive for BCL-2. Intensity and extent scores were multiplied to generate an H-score ranging from 0 to 300. Based on previous research [23], we established an H-score of 20 as cut-off to discriminate between BCL-2^−^ and BCL-2^+^ samples.

### 2.4. NGS

DNA was extracted from isolated bone marrow cells using the QIAamp DNA Blood Mini Kit (QIAgen, Hilden, Germany) as per the manufacturer’s instructions. A HaloPlex Target Enrichment kit (Agilent Technologies) was used to produce libraries of exonic regions from 29 genes (*ASXL1*, *BCOR*, *CALR*, *CSF3R*, *CBL*, *CEBPA*, *DNMT3A*, *ETNK1*, *EZH2*, *FLT3*, *IDH1*, *IDH2*, *JAK2*, *KMT2A*, *KIT*, *KRAS*, *MPL*, *NPM1*, *NRAS*, *RUNX1*, *SETBP1*, *SF3B1*, *SRSF2*, *STAG2*, *TET2*, *TP53*, *U2AF1*, *WT1*, and *ZRSR2*) from 50 ng of genomic DNA. Index-tagged libraries were then quantified using an HS Qubit dsDNA assay (Invitrogen, Carlsbad, CA, USA) and pooled in equimolar amounts for paired-end sequencing on an Illumina MiSeq system using a Miseq Reagent Kit (v.2.0; 500 cycles; Illumina, San Diego, CA, USA). Data analysis was performed with SeqNext software (JSI Medical Systems, Ettenheim, Germany). Additionally, we used Pindel (https://www.sanger.ac.uk/science/tools/pindel) to detect large *FLT3* indels. *FLT3* variants of inconclusive pathogenicity were considered negative. A threshold of 2% of mutated DNA in a wild-type background was applied to ensure a sensitivity of 5%. Regions covered by <500 reads did not fulfill the analysis requirements and were considered uninformative.

### 2.5. Clinical Outcome Evaluation

Patients who received at least one cycle of IC and at least four cycles of HMAs were considered evaluable for response. Treatment response was defined as any patient under IC or HMAs who obtained a complete remission (CR), CR with incomplete blood recovery (CRi), CR with incomplete platelet recovery (CRp), morphologic leukemia-free state (MLFS), or partial remission (PR) on bone marrow re-evaluation. We used the definitions of CR, CRi, CRp, MLFS, and PR as detailed in the 2017 ELN recommendations [1]. Patients who were primary refractory or experienced an early death (defined as death during IC or within the first four HMA cycles) were categorized as non-responders. OS was calculated from time of diagnosis until date of last follow-up or death from any cause.

### 2.6. Statistical Analyses

Statistical analysis and graphing were performed using SPSS (version 26.0; IBM Corp., Armonk, NY, USA) and/or GraphPad Prism (version 8.0.2; GraphPad Software, La Jolla, CA, USA). The results from descriptive statistics were reported as frequencies or mean values ± standard errors of the mean (SEM). Contingency analysis with Fisher’s exact test was performed for categorical variables. For continuous variables, Mann–Whitney (non-parametric) or unpaired Student’s *t*-tests (parametric) were used depending on whether the variables failed or passed the Kolmogorov–Smirnov normality test, respectively. Kaplan−Meier OS curve comparison was performed using the Gehan–Breslow–Wilcoxon test. A *p*-value < 0.05 was considered statistically significant.

## 3. Results

### 3.1. BCL-2 Is Heterogeneously Expressed in AML

**Figure 1:**
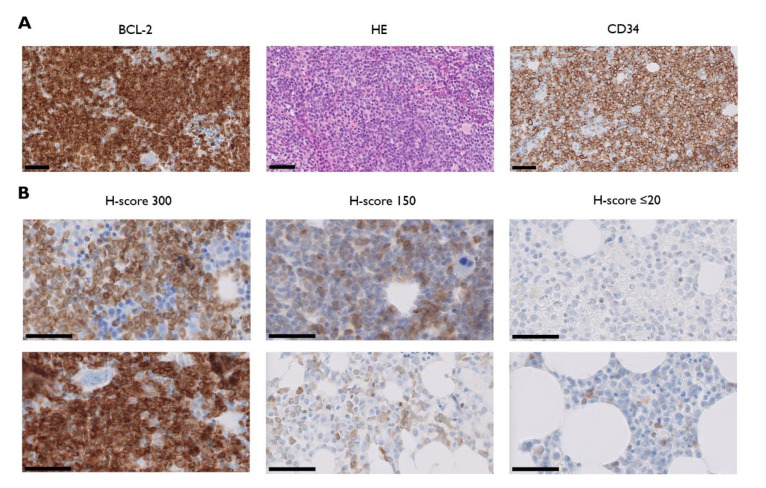
(**A**) Light micrographs of BCL-2 (B-cell leukemia/lymphoma-2, left) and corresponding haematoxylin and eosin (HE; middle) and CD34 stained paraffin sections of trephine biopsies from one representative acute myeloid leukemia patient with a BCL-2 H-score of 300; (**B**) bone marrow BCL-2 immunostains from two representative patients with H-scores of 300 (left panel), 150 (middle panel), and ≤20 (right panel). Thick black line = 50 µm.

We collected data from 112 patients consecutively diagnosed with and/or treated for AML between 1 January 2017 and 31 December 2019, at the Antwerp University Hospital, a large, tertiary academic center in Belgium (Table 1). Ninety-three patients received a trephine biopsy at diagnosis and were evaluable for BCL-2 protein analysis by IHC. BCL-2 was overexpressed in 72 of 93 cases (77.4%), whereas 21 of 93 samples (22.6%) showed no or negligible expression (defined as absent or weak expression in ≤20% of AML blasts). BCL-2 staining intensity in BCL-2^+^ patients was highly heterogeneous, with H-scores ranging from 20 to 300 (Figure 1).

As shown in Table 1, there were no statistically significant differences in baseline patient demographics (age or gender) between the BCL-2^−^ and BCL-2^+^ subgroups nor were there differences in AML diagnosis (de novo AML vs. secondary AML) or AML risk profile (favorable, intermediate, and adverse). There was also an equal frequency distribution of the four main WHO subtypes (i.e., AML with recurrent genetic abnormalities, AML with myelodysplasia-related changes, therapy-related AML, and AML not otherwise specified [18]) among the total patient population and the BCL-2^−^/BCL-2^+^ subgroups. AML with mutated *NPM1* was the most common type within the “AML with recurrent genetic abnormalities” category, occurring in 24% of all AML cases and in 23.8% and 23% of BCL-2^−^ and BCL-2^+^ AML cases, respectively.

### 3.2. BCL-2^−^ and BCL-2^+^ AML Subgroups Show Similar Proliferative Activity but Different Bone Marrow Blast Percentages

To assess BCL-2 as a marker of proliferative activity in AML subgroups, we compared the degree of leukocytosis, as well as the presence/absence of peripheral blasts, between the BCL-2^−^ and BCL-2^+^ subgroups. Despite a lower absolute value, there was no statistically significant difference in mean WBC count in the BCL-2^−^ subgroup as compared with the BCL-2^+^ subgroup (24.9 ± 8.5 × 10^9^/L vs. 36.3 ± 7.0 × 10^9^/L; *p* = 0.7793). Additionally, six of 21 (28.6%) BCL-2^−^ AML patients showed a WBC count of ≥25 × 10^9^/L, an important marker of proliferative disease [24], as compared with 24 of 72 patients (33.3%) in the BCL-2^+^ subgroup. Similarly, peripheral blasts were detected in 90.5% and 87.3% of BCL-2^−^ and BCL-2^+^ patients, respectively. In contrast, the mean bone marrow blast percentage was significantly lower in the BCL-2^−^ subgroup as compared with the BCL-2^+^ subgroup (42.1 ± 5.1% vs. 54.9 ± 3.1%; *p* = 0.0472). This difference was most pronounced in patients with favorable and adverse ELN/cytogenetic risk (*p* = 0.0128), whereas there was no statistically significant difference in mean bone marrow blast percentage in the intermediate risk group. Although a previous study established a correlation between BCL-2 expression and CD34 expression [25], our results showed that BCL-2 is not differentially expressed among CD34^−^ and CD34^+^ AML patients. The mean BCL-2 H-score among the 27 evaluable patients with CD34^−^ AML was 104 ± 16 versus 125 ± 11 among the 61 CD34^+^ AML patients (*p* = 0.2990). There was a clear trend (*p* = 0.0859) towards lower BCL-2 expression in patients with monocytic AML, defined here on the basis of CD64 positivity [26]. The mean BCL-2 H-score among the 22 evaluable CD64^+^ AML patients was 89 ± 17 compared to 126 ± 12 in the 55 evaluable CD64^−^ patients. The difference was even more pronounced in the 11 evaluable patients with strong CD64 expression, as evidenced by the mean BCL-2 H-score of 78 ± 22 in this specific subgroup.

### 3.3. BCL-2^−^ AML Subgroup Is Characterized by a Distinct Molecular Signature

**Figure 2:**
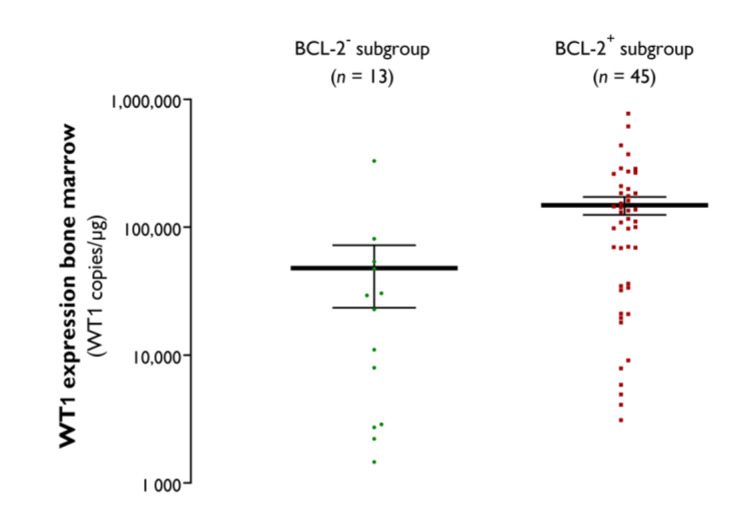
Bone marrow *WT1* transcript levels in the BCL-2^−^ and BCL-2^+^ AML subgroups, with the black horizontal lines indicating the mean ± SEM values (copies/µg).

The *Wilms’ tumor 1* (*WT1*) gene, whose protein product acts as a transcription factor, regulating cell development and survival and whose quantitative assessment is a useful tool to measure disease burden in AML [14,27,28], was overexpressed in either the peripheral blood or bone marrow in 17 of 19 (89.5%) evaluable BCL-2^−^ AML patients and in 57 of 60 (95.0%) evaluable BCL-2^+^ AML patients. In line with the lower bone marrow blast percentage, the *WT1* transcript burden in the bone marrow was significantly lower in the BCL-2^−^ AML subgroup (47,910 ± 24,410 copies/µg) as compared with the BCL-2^+^ subgroup (148,900 ± 23,700 copies/µg; *p* = 0.0016) (Figure 2).

*FLT3* PCR fragment analysis was performed in 19/21 patients in the BCL-2^−^ AML subgroup. Only one *FLT3*-ITD was detected and none of the patients had a TKD mutation. In the BCL-2^+^ subgroup, *FLT3* mutational status was assessed by conventional RT-PCR in 61/72 patients. Sixteen of these patients had a *FLT3*-ITD mutation, one had a *FLT3*-TKD mutation, and two were both *FLT3*-ITD and *FLT3*-TKD positive. The prevalence of *FLT3* mutations in the BCL-2^−^ subgroup (1/19; 5.3%) was significantly lower relative to that in the BCL-2^+^ subgroup (19/61; 31.1%) (*p* = 0.0314).

Molecular profiling by NGS confirmed the underrepresentation of *FLT3* mutations in the BCL-2^−^ subgroup (Table 2). No other statistically significant differences in NGS profiles were identified except for a trend towards dominance of *NRAS* mutations in the BCL-2^+^ subgroup (Table 2). Of the BCL-2^−^ patients, 88.9% harbored at least one somatic alteration (range: 1–7; mean: 3), which was comparable with their BCL-2^+^ counterparts (84.6%; range: 1–7; mean: 3). The mutational burden in the BCL-2^−^ subgroup (45/524; 8.6%) was identical to that in the BCL-2^+^ subgroup (154/1785; 8.6%).

### 3.4. Absence of BCL-2 Expression Identifies a Subgroup of AML Patients with Good Response to Chemotherapy and Favorable OS

In the BCL-2^−^ subgroup, 71.4% of the patients received IC and 19.0% HMAs. This was comparable with the BCL-2^+^ subgroup, which was composed of 62.5% IC-treated patients and 22.2% HMA-treated patients. The overall response rate (CR + CRi + CRp + MLFS + PR) to IC and HMAs was 94.1% (16 of 17 response-evaluable patients) in the BCL-2^−^ subgroup, whereas it was only 61.0% (36 of 59 patients) in the BCL-2^+^ subgroup (*p =* 0.0086).

**Figure 3:**
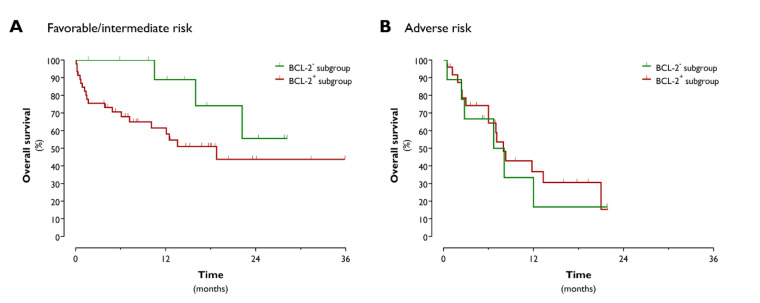
Kaplan–Meier overall survival curves of favorable/intermediate (**A**) and adverse (**B**) risk BCL-2^−^ and BCL-2^+^ AML patients.

The median OS tended to be prolonged in the BCL-2^−^ subgroup, with nearly a doubling of the OS time for IC-treated BCL-2^−^ AML patients as compared to their BCL-2^+^ counterparts (22.2 months vs. 13.3 months; *p* = 0.0778). Given the underrepresentation of *FLT3* mutations in the BCL-2^−^ subgroup and the known negative prognostic impact of *FLT3*-ITD, we next aimed to determine whether the observed difference in median OS was due to the different *FLT3*-ITD mutational statuses between the two subgroups. Exclusion of the *FLT3*-ITD^+^ patients had no impact on the median OS difference between the IC-treated BCL-2^−^ and BCL-2^+^ patients (22.2 months vs. 10.1 months; *p* = 0.0690). The OS advantage was clearly demonstrable in favorable/intermediate risk BCL-2^−^ AML patients (median OS of BCL-2^−^ vs. BCL-2^+^ patients: undefined vs. 18.8 months; *p* = 0.0411; Figure 3A). The median OS of adverse risk BCL-2^−^ patients was 7.4 months compared to 8.0 months in the BCL-2^+^ cohort (*p* = 0.6040; Figure 3B).

## 4. Discussion

In recent years, the anti-apoptotic protein BCL-2 has emerged as an attractive therapeutic target in a variety of cancers, including AML [5]. The oral BCL-2 inhibitor venetoclax has shown strong clinical activity in AML patients, in particular when combined with conventional chemotherapies [6,13]. The addition of venetoclax to standard therapy will likely become the new treatment paradigm in AML in the very near future [5]. For pharmacoeconomic reasons and because of the risk of serious additive toxicities, it is imperative to identify which AML patients will benefit most from the incorporation of venetoclax into their treatment regimen. Here, we report on bone marrow BCL-2 IHC as a simple, rapid, and low-cost method to identify a subpopulation of AML patients with no or negligible BCL-2 protein expression. This group displayed a low-risk profile on the phenotypic, molecular, and clinical level, with good response to standard therapy, thus precluding the need for the addition of venetoclax.

There is a general assumption that BCL-2 is broadly expressed among AML patients. Our study, which comprised a representative group of AML patients with cytogenetic and molecular characteristics that conformed to real-world data [29], however, indicates that 1 in 5 AML patients should be considered as BCL-2^−^ and that there is a strong inter-patient heterogeneity, both in terms of BCL-2 staining intensity and frequency, among the BCL-2^+^ patients. Using the same cut-off for positivity as applied in the current study, Campos et al. [23] previously demonstrated BCL-2 positivity in only 34% of newly diagnosed AML patients. Our results are more in line with the study by Bensi et al. [30], who found evidence of BCL-2 expression (defined by the same 20% cut-off level) in 68.3% of the cases at diagnosis.

Previous work has linked BCL-2 expression in AML with distinct phenotypic features. For example, BCL-2 expression was found to correlate with higher WBC counts at diagnosis [23], a finding that could not be substantiated in the present study. Likewise, we were not able to demonstrate a statistically significant difference in BCL-2 protein expression level between CD34^+^ and CD34^−^ AML patients, whereas others have shown that BCL-2 is maximally expressed in the CD34^+^ leukemic cell compartment, followed by a downregulation upon the loss of CD34 during differentiation [25,31,32]. In line with two recent studies demonstrating lower *BCL-2* gene expression levels in AMLs with a monocytic component [33,34], we observed lower BCL-2 protein levels in monocytic AML. This might, at least in part, explain why AMLs with a monocytic phenotype tend to be more resistant to venetoclax-based therapy. Again at the phenotypic level, this study revealed a higher percentage of bone marrow blasts in patients with demonstrable BCL-2 expression. We believe that the prognostic relevance of this finding is negligible, given the fact that only AML with a low blast count (i.e., 20–29%) is associated with a more favorable prognosis; in AML with ≥30% blasts—as was the case for both the BCL-2^−^ and BCL-2^+^ subgroups—the prognostic impact of the bone marrow blast percentage at baseline has not been clearly demonstrated [24].

At the molecular level, the BCL-2^−^ AML subgroup was clearly marked by a favorable prognostic profile. Consistent with a previous study by Karakas et al. [35], who observed a correlation between *WT1* and *BCL-2* transcript levels in the bone marrow, we detected lower peripheral blood and bone marrow *WT1* expression levels in BCL-2^−^ patients. Several studies have shown that *WT1* overexpression is a poor independent prognostic marker in AML, in particular in cytogenetically normal AML [35,36,37]. The lower *WT1* expression in the BCL-2^−^ AML subgroup can thus be considered as an indicator of a more favorable prognosis. In addition, whereas the NGS mutational profile of the BCL-2^+^ group conformed to real-world data [29], the BCL-2^−^ subgroup displayed an underrepresentation of *FLT3* (5.3%) and *NRAS* (0%) mutations. The anticipated frequency of these mutations in AML is ~30% and 15–20%, respectively [29]. Although the prognostic relevance of *(N)RAS* mutation remains debatable [38,39,40,41], it is well established that the presence of *FLT3*-ITD, which is the most common *FLT3* mutational variant, is a poor prognostic factor [42,43]. However, detailed analysis revealed that the observed OS benefit in BCL-2^−^ patients was not due to the lower frequency of *FLT3*-ITD mutations. In line with a previous study from the pre-molecular era by Campos et al. [23], this indicates that bone marrow BCL-2 protein expression serves as a prognostic marker independent of the molecular profile. Importantly, the prognostic value of BCL-2 positivity was only demonstrable in patients with favorable or intermediate ELN/cytogenetic risk. Hence, for these patients, determination of the baseline bone marrow BCL-2 protein expression can be helpful to improve the current risk stratification and make informed decisions about the need to proceed to allogeneic hematopoietic stem cell transplantation in first CR.

In this study, IC or HMAs alone appeared to be sufficient to induce a CR in the BCL-2^−^ subgroup. Based on this observation, a standard chemotherapy approach (IC or HMAs) alone is a rational choice of therapy in AML patients with non-detectable or marginally detectable bone marrow BCL-2 protein expression. We believe that the added value of venetoclax is highly questionable in this particular patient population. This, however, does not preclude the possibility that venetoclax might have therapeutic activity even in patients without apparent BCL-2 expression. It is becoming increasingly clear that the mutational profile at baseline is predictive of the subsequent response to anti-BCL-2 therapy [12]. For example, single agent venetoclax [11] and venetoclax used in combination with chemotherapy [6,8,12,13] has high therapeutic activity in newly diagnosed AML patients carrying *NPM1* and *IDH2* mutations. In the present study, we observed an equal distribution of *NPM1* and *IDH2* mutations between BCL-2^−^ and BCL-2^+^ AML patients, suggesting that BCL-2 positivity is not a prerequisite for obtaining a response to venetoclax therapy.

To conclude, absent or low BCL-2 protein expression in the bone marrow identifies a subgroup of AMLs with a lower bone marrow blast percentage, a favorable molecular profile and good response to standard therapies, translating into prolonged survival. We believe that the high drug costs of venetoclax and its potential to induce greater toxicity does not legitimize the addition of venetoclax in this AML subgroup. This group, which represents approximately one in five newly diagnosed AML patients, was determined according to negative BCL-2 expression through IHC analysis on trephine biopsy obtained at diagnosis. This fast and inexpensive test can be easily applied and used in the future to rationalize the choice of initial treatment in AML, potentially avoiding unnecessary add-on therapy with venetoclax.

## Figures and Tables

**Table 1:** Baseline characteristics of the BCL-2^−^ and BCL-2^+^ AML subgroups.

	All Patients	BCL-2^−^	BCL-2^+^
		H-Score 0–20	H-Score 21–300
**Number (%)**	112	21 (22.6%)	72 (77.4%)
**Median age in years (range)**	65 yr (19–92 yr)	67 yr (24–87 yr)	67 yr (19–89 yr)
**Male (%)**	60 (53.6%)	10 (47.6%)	42 (58.3%)
**Female (%)**	52 (46.4%)	11 (52.4%)	30 (41.7%)
**De novo AML (%)**	78 (69.6%)	14 (66.7%)	49 (68.1%)
**Secondary AML (%)**	34 (30.4%)	7 (33.3%)	23 (31.9%)
**ELN/Cytogenetic risk**			
Favorable	26 (25.0%)	7 (33.3%)	14 (19.7%)
Intermediate	41 (39.4%)	5 (23.8%)	32 (45.1%)
Adverse	37 (35.6%)	9 (42.9%)	25 (35.2%)

BCL-2, B-cell leukemia/lymphoma-2; AML, Acute myeloid leukemia.

**Table 2:** Comparison of NGS profiles between the BCL-2^−^ and BCL-2^+^ AML subgroups.

	BCL-2^−^	BCL-2^+^	*p*-Value
	(H-Score 0-20)	(H-Score 21-300)	
**DNA methylation**			
*DNMT3A*	5 (27.8%)	15 (24.6%)	>0.05
*TET2*	4 (22.2%)	16 (26.2%)	>0.05
*IDH1*	1 (5.6%)	6 (9.8%)	n.d.
*IDH2*	3 (16.7%)	5 (8.2%)	>0.05
**Tumor suppressor genes**			
*TP53*	3 (16.7%)	9 (14.8%)	n.d.
*WT1*	0 (0.0%)	6 (9.8%)	>0.05
**Epigenetic modifiers**			
*ASXL1*	3 (16.7%)	3 (4.9%)	>0.05
*BCOR1*	2 (11.1%)	7 (11.5%)	n.d.
*EZH2*	0 (0.0%)	1 (1.6%)	n.d.
*KMT2A*	0 (0.0%)	0 (0.0%)	n.d.
**Transcription factors**			
*CEBPA*	0 (0.0%)	6 (9.2%)	n.d.
*RUNX1*	4 (22.2%)	9 (14.8%)	>0.05
*SETBP1*	0 (0.0%)	0 (0.0%)	n.d.
**Nucleophosmin 1**			
*NPM1*	6 (31.6%)	18 (27.7%)	>0.05
**Activated signaling**			
*CBL*	0 (0.0%)	0 (0.0%)	n.d.
*FLT3*	1 (5.6%)	20 (30.8%)	=0.0331
*JAK2*	1 (5.6%)	4 (6.6%)	n.d.
*KIT*	0 (0.0%)	3 (4.6%)	n.d.
*KRAS*	4 (22.2%)	1 (1.6%)	n.d.
*MPL*	1 (5.6%)	0 (0.0%)	n.d.
*NRAS*	0 (0.0%)	10 (16.4%)	=0.0626
**RNA Splicing**			
*SF3B1*	1 (5.6%)	4 (6.6%)	n.d.
*SRSF2*	3 (16.7%)	4 (6.6%)	>0.05
*U2AF1*	1 (5.6%)	2 (3.3%)	n.d.
**Cohesin complex**			
*STAG2*	2 (11.1%)	4 (6.6%)	n.d.
**Other**			
*CALR*	0 (0.0%)	1 (1.6%)	n.d.
*CSF3R*, *ETNK1* and *ZRSR2*	0 (0.0%)	0 (0.0%)	n.d.

NGS, Next-generation sequencing; n.d., not determined; testing for statistical significance was only determined for driver gene mutations with an anticipated frequency of >10% according to Metzeler et al. [29].
